# Harnessing Polygenic Risk Scores to Refine Venous Thromboembolism Risk Stratification

**DOI:** 10.1111/ejh.70212

**Published:** 2026-05-07

**Authors:** Katlyn G. McKay, Oliver S. Zhao, Samaya S. Badrieh, Lisa Bastarache, Xinnan Niu, Jing He, Prince J. Kannankeril, Jamie R. Robinson

**Affiliations:** ^1^ Section of Surgical Sciences Vanderbilt University Medical Center Nashville Tennessee USA; ^2^ Vanderbilt University School of Medicine Nashville Tennessee USA; ^3^ Information School University of Washington Seattle Washington USA; ^4^ Department of Biomedical Informatics Vanderbilt University Medical Center Nashville Tennessee USA; ^5^ Department of Pediatrics, Center for Pediatric Precision Medicine Vanderbilt University Medical Center Nashville Tennessee USA; ^6^ Department of Pediatric Surgery Vanderbilt University Medical Center Nashville Tennessee USA

**Keywords:** genetic variants, polygenic risk score, venous thromboembolism

## Abstract

**Background:**

Venous thromboembolism (VTE) is a major cause of morbidity in patients of all ages. Despite growing interest in polygenic risk scores (PRS) for VTE, their utility remains understudied. Our objective was to evaluate the independent impact of a PRS on VTE susceptibility in adults and children.

**Methods:**

We completed a retrospective, case–control study of two separate cohorts with evaluation of three VTE PRS models, with the primary analysis focused on a 293 single nucleotide polymorphism (SNP) PRS. The adult cohort included 597 VTE cases and 31 998 controls, and the pediatric cohort included 109 cases and 448 controls, both obtained from a de‐identified databank with linked genetic data. Separate adult and pediatric multivariable logistic regressions were performed to measure the association of risk factors with VTE.

**Results:**

Higher PRS in adults was significantly associated with increased odds of VTE, with each 1‐standard deviation increase in PRS conferring an adjusted odds ratio of 1.25 (OR = 1.25, 95% CI 1.15–1.36, *p* < 0.001). Leading risk factors for adults were cancer (OR = 2.43, 95% CI: 2.04–2.89, *p* < 0.001) and recent surgery (OR = 2.16, 95% CI: 1.83–2.54, *p* < 0.001). The standardized PRS also exhibited increased risk for VTE in children (OR = 1.38, 95% CI 1.10–1.74, *p* = 0.003). Central venous catheterization (OR = 5.65, 95% CI 3.40–9.50, *p* < 0.001) was the foremost risk factor for pediatric VTE.

**Conclusion:**

VTE in adults and children is multifactorial, with clinical and genome‐wide risk factors contributing. PRS may serve as a valuable adjunct to clinical risk factors for VTE risk stratification.

## 1 Introduction

Venous thromboembolism (VTE), including deep venous thrombosis and pulmonary embolism, is a major public health issue affecting over 1 in 1000 adults worldwide annually [1, 2]. While more rare in children, the VTE incidence in hospitalized children has increased 10‐fold over the last 20 years with a current estimated rate of 1 in 200 pediatric admissions [3, 4, 5]. Effective prophylaxis measures such as pharmacological agents, mechanical devices such as sequential compression devices, and early mobilization protocols in higher‐risk patient populations can reduce the risk of VTE and prevent subsequent morbidity or mortality [6]. Tailoring prophylactic strategies based on individual risk profiles is crucial for optimizing patient care and outcomes. Factors such as surgery, prolonged immobility, trauma, and cancer are among the most previously reported clinical risk factors for VTE in adults [7, 8, 9]. Central venous catheters (CVCs) for patients requiring long‐term venous access is also a known risk factor for VTE in adults and children. However, the prevalence of CVC‐related thrombosis in children has been shown to be much higher than in adults, associated with 50%–80% of pediatric VTEs compared to 10% in adults [10, 11, 12, 13]. Other risk factors for VTE in pediatric patients include trauma, malignancy, and renal diseases [14].

In addition to clinical risk factors, almost 25% of VTE incidence can be attributed to genomic risk, suggesting a potential role for genomic information to direct prediction and prophylaxis for VTE [15, 16]. Genome‐wide polygenic risk scores (PRSs) have shown success in improving prediction of a variety of diseases, including obesity, cancer, cardiometabolic traits, stroke, and others [17, 18, 19, 20]. For VTE, prior meta‐analyses in adult populations have demonstrated that a PRS of 135 VTE‐associated loci in Europeans was associated with a 6‐fold increase in risk for those in the top 1% of polygenic risk compared to those with average risk scores [21]. Other studies have demonstrated that the top 0.1% of a PRS distribution for VTE in adults can have risk similar to homozygous carriers of Factor V Leiden [22]. The impact of genomic risk for VTE in children is far less understood. We sought to better understand the contributing clinical and genetic factors of patients diagnosed with VTE using a broader PRS in both adult and pediatric populations at our institution. We hypothesized that genetic risk will be a significant contributing factor for VTE in both adult and pediatric patients.

## 2 Methods

### 2.1 VTE Cohort Phenotyping

We conducted a single‐institution, retrospective study using the Vanderbilt University Medical Center (VUMC) Synthetic Derivative, a de‐identified version of over 3 million VUMC patient health records dating back several decades and linked to BioVU, VUMC's biorepository of DNA extracted from blood [23, 24]. This study was reviewed by the Institutional Review Board at VUMC (IRB 222072).

#### 2.1.1 Adult Cohorts

**FIGURE 1:**
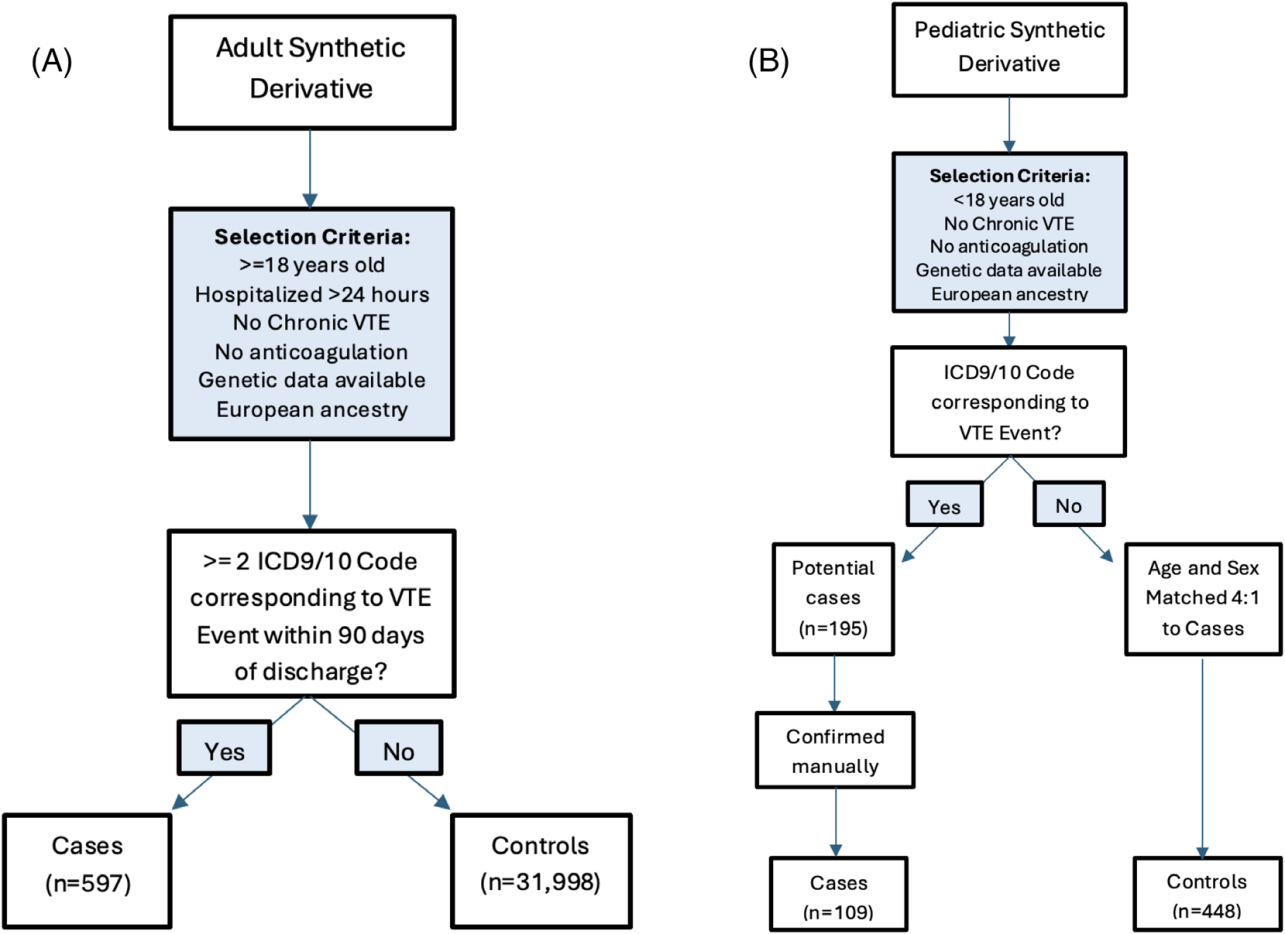
Selection process of venous thromboembolism (VTE) cases and controls for adult (A) and pediatric (B) cohorts.

The adult case and control cohorts consisted of adult patients (≥ 18 years old) with an inpatient hospitalization greater than 24 h and no history of chronic VTE or long‐term anticoagulant therapy based upon International Classification of Diseases (ICD) 9 and 10 codes (ICD9 codes V12.51, V58.61 and ICD10 codes Z86.718, Z79.01, Table S1). Each patient hospital admission was then labeled as either a case or control based on the occurrence of a VTE ICD code within 90 days. Each patient was counted as either a case (at least one hospitalization with a VTE event) or a control (at least one hospitalization without a VTE event and no VTE hospitalizations). To optimize accuracy and positive predictive value, cases had at least two ICD9 or ICD10 codes corresponding to a VTE event within 90 days of hospital discharge. We compared these identified cases to controls without a VTE event who also underwent hospital admission (Figure 1). We extracted covariates with potential VTE association including age at admission, sex, body mass index, history of cancer based upon ICD codes, surgical intervention during the hospitalization based upon CPT codes, and smoking status. Genetic data was derived from BioVU. Individuals of European ancestry with extant genotyping on the Multiethnic Genotyping Array for creation of the PRS were included in the final case and control cohorts.

#### 2.1.2 Pediatric Cohorts

Secondary to overall lower incidence of VTE events in the pediatric population compared to adults, the cases and control cohorts were derived with differing methods. The pediatric population was not limited to VTE events with a recent history of hospitalization to capture a greater patient population. The pediatric case cohort consisted of all children (< 18 years of age) with at least one ICD9 or ICD10 code corresponding to a VTE event (Figure 1). As the cohort was smaller and at greater risk should cases not be identified correctly, all potential cases were manually reviewed by a physician content expert for inclusion. The control cohort was similarly derived using individuals without an ICD code corresponding to a VTE event. Patients were excluded from the control cohort if their medical records had codes indicating chronic venous occlusion or anticoagulant use (ICD9 codes V12.51, V58.61 and ICD10 codes Z86.718, Z79.01, Table S1). We employed a case–control design with approximately 1:4 matching of VTE cases to controls based on age and sex. This approach intentionally enriches the proportion of VTE cases relative to the underlying population incidence to improve statistical power for detecting associations; therefore, the observed differences in VTE proportions across PRS strata reflect relative differences in genetic susceptibility rather than absolute risk. Covariates of CVC insertion and history of a cancer diagnosis were also extracted. Genetic data was derived from BioVU and individuals of European ancestry with extant genotyping on the Multiethnic Genotyping Array for creation of the PRS were included in the final case and control cohorts.

### 2.2 Construction of the PRS

**FIGURE 2:**
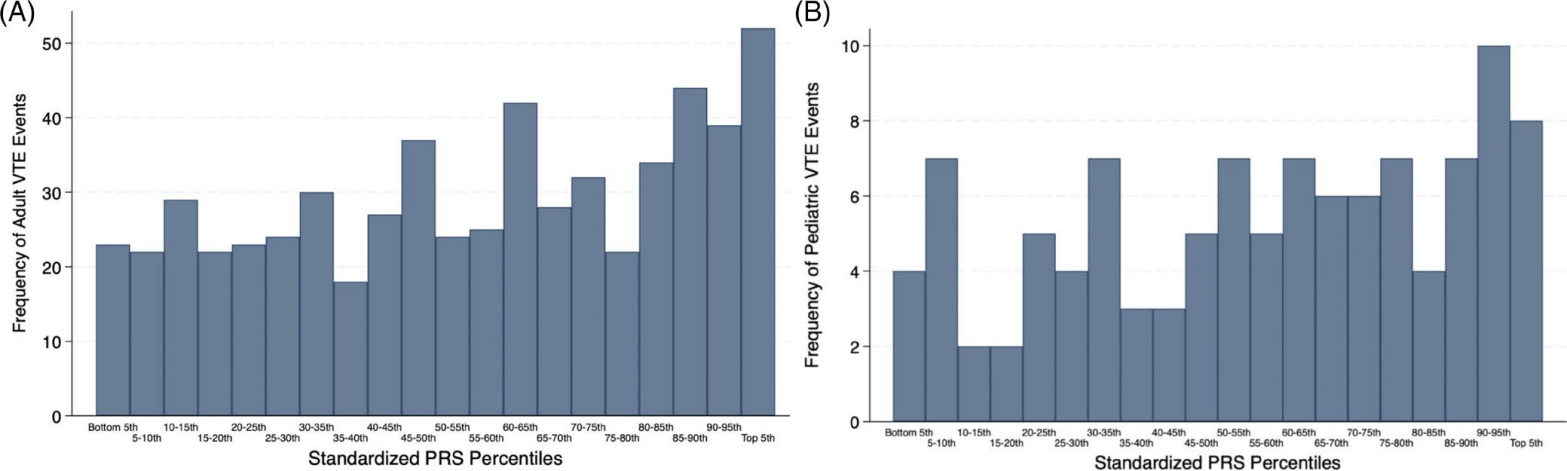
Frequency of venous thromboembolism (VTE) events in adult (A) and pediatric (B) patients stratified by standardized polygenic risk score (PRS) percentiles.

We utilized genome‐wide association study summary statistics from individuals of European ancestry to generate the PRS for each individual [25]. To ensure appropriate interpretation of the PRS created from those of European ancestry, the cohorts above were also limited to those of European ancestry. We calculate three separate PRS for each individual. The first PRS was calculated from beta coefficients of 293 single nucleotide polymorphisms (SNP) associated with VTE at genome‐wide significance previously to estimate individual genetic risk for VTE within the UK Biobank [20]. The second was from a 91‐SNP PRS calculated from available beta coefficients of SNP associated with VTE at genome‐wide significance from a more recent GWAS by Ghouse et al. in 2023 [22]. Factor V Leiden and Factor II variants are present within the 91‐SNP model, and Factor V variants are present in the 293‐SNP model [20, 22]. For both the 293‐SNP and 91‐SNP models, PRS were constructed using two approaches: (1) a summative score (sum of risk allele dosages weighted by effect estimates) and (2) an allelic average (sum of weighted risk allele dosages averaged by the number of successfully genotyped SNPs per individual) to account for variable SNP availability. To assess the independent contribution of Factor V and F2 variants, we calculated a third PRS consisting of only SNP within Factor V and II within the most recent GWAS [22]. PRS and principal component analysis using the first 10 principal components was performed to determine genetic ancestry using PLINK [26]. For the adult and pediatric population cohorts separately, each individual PRS was standardized by transformation to the polygenic Z‐score by taking each individual raw PRS and subtracting the mean PRS in the population followed by division using the standard deviation (SD) of the PRS in the population. This PRS Z‐score indicates how many SDs an individual's score is above or below the average for the population, making the score more easily utilized as a measure of relative risk. These standardized polygenic scores were then categorized into quantiles separately for the adult and pediatric populations (Figure 2). Given differences in SNP availability across cases and controls, the allelic average approach was selected for the 91‐SNP model, and the summative approach was retained for the 293‐SNP model.

### 2.3 PRS Evaluation

A risk model with regression analysis was performed to understand the relative contributions of different covariates, including the three PRS as described above, in predicting VTE risk among adult and pediatric patients. The adult models were adjusted for covariates including the first 10 principal components, age at admission, sex, body mass index, history of cancer, history of recent surgery, and smoking status. The pediatric models were adjusted for covariates relevant for the pediatric population including the first 10 principal components, age, sex, CVC insertion, and history of a cancer diagnosis [27]. CVC insertion was determined based upon CPT code and history of a cancer diagnosis was based upon two instances of an ICD code corresponding to any cancer diagnosis on separate dates. Due to sample size considerations and the need to preserve statistical power, time era was not included as a covariate in regression models. However, several clinical covariates for both cohorts associated with VTE risk were included to partially account for temporal changes in patient characteristics and exposures. Odds ratios (ORs) and their corresponding confidence intervals (CIs) were determined and analyzed for each covariate. The relative proportion of cases of VTE were assessed by ventiles of PRS values in the 293 SNP PRS to determine effect of the extremes of PRS on the population. We then performed a multivariable logistic regression across PRS quantile groups with clinical covariates to determine adjusted ORs. Analyses were performed using R (version 4.3.0; R Core Team, Vienna, Austria, https://www.r‐project.org) [28].

## 3 Results

### 3.1 Adult Risk Model for VTE

**FIGURE 3:**
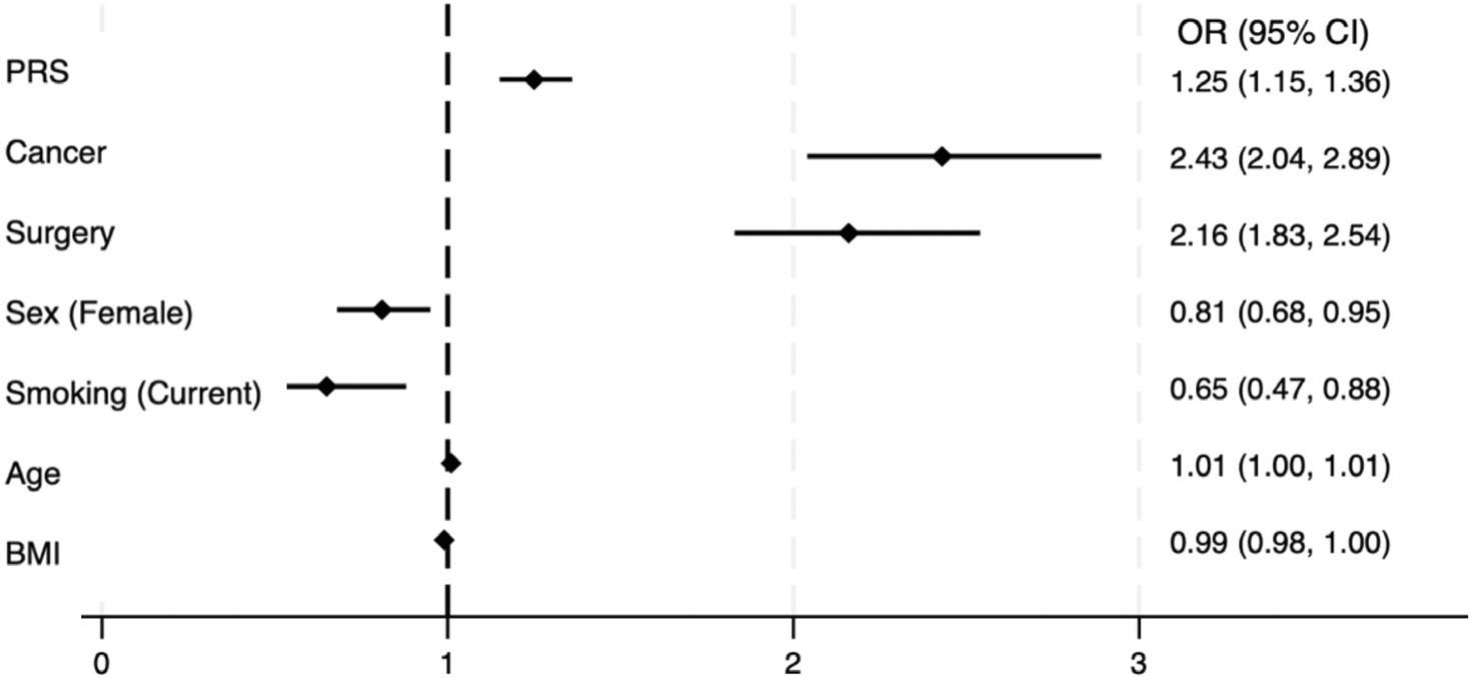
Odds ratios for predictors of venous thromboembolism (VTE) in the adult cohort. Forest plot of odds ratios (OR) with 95% confidence intervals (CI) for predictors of venous thromboembolism (VTE) in the adult cohort. Point estimates represent ORs from multivariable logistic regression with a 293‐SNP model, with horizontal lines indicating 95% CIs.

A total of 597 adult patients with recent hospital inpatient admissions were identified as VTE cases occurring within 90 days. These were compared to 31 998 control patients with hospital admission and no history of subsequent VTE event (Table 1). The average age of patients in the case group was higher than controls (57.2 vs. 53.2 years, *p* < 0.001) and cases were predominantly male at 53.4% (*N* = 319) compared to controls which were 44.9% male (*N* = 14 371, *p* < 0.001). For adults who had a VTE event following hospitalization, 47.1% (*N* = 281) underwent surgery during their hospitalization, compared to 31.3% (*N* = 10 023, *p* < 0.001) of the control group and 56.1% (335) carried a cancer diagnosis compared to 34.0% of controls (10 864, *p* < 0.001, Table 2). Multivariable logistic regression was performed with three PRS models. The standardized PRS with 293 SNPs was significantly associated with increased odds of VTE after adjustment for age, sex, cancer, smoking, and principal components (OR = 1.25, 95% CI: 1.15–1.36, *p* < 0.001). Cancer (OR = 2.43, 95% CI: 2.04–2.89, *p* < 0.001) and recent surgery (OR = 2.16, 95% CI: 1.83–2.54, *p* < 0.001) were the strongest clinical predictors of VTE in the adult cohort (Figure 3). The 91‐SNP model demonstrated similar results, with a significant association between standardized PRS and VTE risk (OR 1.33, 95% CI 1.23–1.43, *p* < 0.001), with cancer (OR 2.44, 95% CI 2.05–2.91, *p* < 0.001) and surgery (OR 2.16, 95% CI 1.84–2.55, *p* < 0.001) demonstrating the strongest clinical prediction (Table 3). The simplified 2‐SNP model incorporating only Factor V Leiden and Factor II variants did not demonstrate a significant association with VTE risk (OR 0.90, 95% CI 0.69–1.13, *p* = 0.409).

**TABLE 1:** Baseline characteristics of pediatric and adult cohorts stratified by polygenic risk score (PRS) percentile group using a 293‐SNP PRS.

Pediatric cohort	Bottom 10th percentile PRS	Middle 80th percentile PRS	Top 90th percentile PRS	*p*
	*N* = 56	*N* = 446	*N* = 55	
Age in years, mean (SD)	6.97 (6.80)	6.39 (6.64)	7.47 (6.78)	0.46
Male, *n* (%)	25 (44.6%)	231 (51.8%)	28 (50.9%)	0.60
Presence of central line, *n* (%)	15 (26.8%)	82 (18.4%)	9 (16.4%)	0.28
Cancer diagnosis, *n* (%)	2 (3.6%)	11 (2.5%)	2 (3.6%)	0.80
VTE cases, *n* (%)	11 (19.6%)	80 (17.9%)	18 (32.7%)	0.03

Adult cohort	*N* = 3260	*N* = 26 076	*N* = 3259	
Age in years, mean (SD)	53.48 (17.41)	53.33 (17.34)	52.85 (17.32)	0.27
Male, *n* (%)	1466 (45.0%)	11 742 (45.0%)	1482 (45.5%)	0.88
Body mass index, mean (SD)	29.13 (7.60)	29.07 (7.49)	29.07 (7.64)	0.90
Cancer diagnosis, *n* (%)	1153 (35.4%)	8961 (34.4%)	1085 (33.3%)	0.21
Recent surgery, *n* (%)	1074 (32.9%)	8195 (31.4%)	1035 (31.8%)	0.21
Smoking, *n* (%)				
Current	365 (11.2%)	2849 (10.9%)	345 (10.6%)	0.69
Never	1436 (44.0%)	11 271 (43.2%)	1435 (44.0%)	
Past	540 (16.6%)	4618 (17.7%)	579 (17.8%)	
Unknown	919 (28.2%)	7338 (28.1%)	900 (27.6%)	
VTE cases, *n* (%)	1.4% (45)	1.8% (461)	2.8% (91)	< 0.001

*Note:* Continuous variables (age, BMI) compared across PRS quantile groups using ANOVA; categorical variables (sex, cancer, surgery, smoking, VTE cases) compared using Chi‐squared tests.

Abbreviations: PRS, polygenic risk score; SNP, single nucleotide polymorphism; SD, standard deviation; VTE, venous thromboembolism.

**TABLE 2:** Baseline characteristics of venous thromboembolism (VTE) cases and controls in pediatric and adult cohorts.

Pediatric cohort	VTE cases	Controls	*p*
	*N* = 109	*N* = 448	
Age in years, mean (SD)	6.22 (6.65)	6.63 (6.68)	0.57
Male, *n* (%)	59 (54.1%)	225 (50.2%)	0.46
Presence of central line, *n* (%)	46 (42.2%)	60 (13.4%)	< 0.001
Cancer diagnosis, *n* (%)	4 (3.7%)	11 (2.5%)	0.48
293‐SNP PRS, mean (SD)	261.43 (13.13)	256.83 (12.22)	< 0.001
Standardized 293‐SNP PRS, mean (SD)	0.23 (1.05)	−0.06 (0.98)	0.01
Standardized 293‐SNP allelic average PRS, mean (SD)	0.18 (0.99)	−0.04 (1.00)	0.04
91‐SNP PRS, mean (SD)	89.84 (5.30)	90.33 (5.82)	0.42
Standardized 91‐SNP PRS, mean (SD)	−0.07 (0.93)	0.02 (1.02)	0.40
Standardized 91‐SNP allelic average PRS, mean (SD)	0.18 (0.92)	−0.04 (1.01)	0.04

Adult cohort	*N* = 597	*N* = 31 998	
Age in years, mean (SD)	57.23 (16.00)	53.22 (17.36)	< 0.001
Male, *n* (%)	319 (53.4%)	14 371 (44.9%)	< 0.001
Body mass index m/kg^2^, mean (SD)	28.38 (7.41)	29.09 (7.52)	0.02
Cancer diagnosis, *n* (%)	335 (56.1%)	10 864 (34.0%)	< 0.001
Recent surgery, *n* (%)	281 (47.1%)	10 023 (31.3%)	< 0.001
Smoking, *n* (%)			
Current	47 (7.9%)	3512 (11.0%)	< 0.001
Never	284 (47.6%)	13, 858 (43.4%)	
Past	139 (23.3%)	5598 (17.5%)	
Unknown	127 (21.3%)	9030 (28.2%)	
293‐SNP PRS, mean (SD)	260.90 (13.22)	258.24 (12.42)	< 0.001
Standardized 293‐SNP PRS, mean (SD)	0.21 (1.06)	−0.004 (1.00)	< 0.001
Standardized 293‐SNP allelic average PRS, mean (SD)	0.23 (1.06)	−0.00 (1.00)	< 0.001
91‐SNP PRS, mean (SD)	90.45 (5.46)	90.17 (5.59)	0.23
Standardized 91‐SNP PRS, mean (SD)	0.05 (0.98)	−0.00 (1.00)	0.23
Standardized 91‐SNP allelic average PRS, mean (SD)	0.28 (1.07)	−0.00 (1.00)	< 0.001

*Note:* Continuous variables (age, BMI, PRS) compared across VTE case and control groups using *t* tests; categorical variables (sex, cancer, surgery, smoking) compared using Chi‐squared tests.

Abbreviations: PRS, polygenic risk score; SD, standard deviation; VTE, venous thromboembolism.

**TABLE 3:** Multivariable logistic regression of risk factors for venous thromboembolism (VTE) in adult population.

Variable	293‐SNP PRS OR (95% CI)	293‐SNP PRS *p* value	91‐SNP PRS OR (95% CI)	91‐SNP PRS *p* value
Age	1.01 (1.00–1.01)	0.03	1.01 (1.00–1.01)	0.03
Female	0.80 (0.68–0.95)	0.012	0.80 (0.68–0.95)	0.01
BMI	0.99 (0.98–1.00)	0.14	0.99 (0.98–1.00)	0.13
Cancer	2.43 (2.04–2.89)	< 0.001	2.44 (2.05–2.91)	< 0.001
Smoking status				
Never	Ref	Ref	Ref	
Past	1.00 (0.81–1.23)	1.00	1.00 (0.81–1.23)	0.98
Current	0.65 (0.47–0.88)	0.01	0.65 (0.47–0.88)	0.01
Unknown	0.55 (0.45–0.69)	< 0.001	0.55 (0.44–0.68)	< 0.001
Surgery	2.16 (1.83–2.54)	< 0.001	2.16 (1.84–2.55)	< 0.001
Standardized PRS	1.25 (1.15–1.36)	< 0.001	1.33 (1.23–1.43)	< 0.001

*Note:* Two multivariable logistic regressions performed (one for 293‐SNP, one for 91‐SNP), adjusted for the first 10 principal components, age, sex, BMI, history of a cancer diagnosis, smoking, and recent surgery.

Abbreviations: BMI, body mass index; CI, confidence interval; OR, odds ratio; PRS, polygenic risk score; SD, standard deviation; SNP, single nucleotide polymorphism; VTE, venous thromboembolism.

For the lowest decile (< 10th percentile) of standardized PRS, there were 45 of 3260 adults (1.4%) with a VTE event. For the highest decile (> 90th percentile) of standardized PRS, there were 91 of 3260 adults (2.8%) with a VTE event (Table 1). Thus, adult individuals with a PRS in the highest decile were twice as likely to have a VTE event than adult individuals with a PRS in the lowest decile. Individuals in the top 10% of PRS distribution had 2.17 times higher odds of VTE compared to those in the bottom 10% (OR 2.17, 95% CI 1.51–3.13, *p* < 0.001), after adjusting for age, sex, cancer, surgery, smoking, and principal components.

### 3.2 Pediatric Risk Model for VTE

**FIGURE 4:**
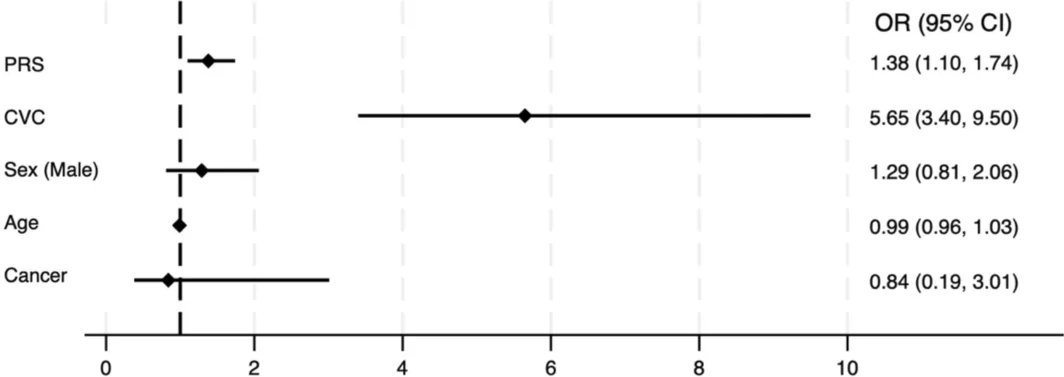
Odds ratios for predictors of venous thromboembolism (VTE) in the pediatric cohort. Forest plot of odds ratios (OR) with 95% confidence intervals (CI) for predictors of venous thromboembolism (VTE) in the pediatric cohort. Point estimates represent ORs from multivariable logistic regression with a 293‐SNP model, with horizontal lines indicating 95% CIs.

A total of 195 pediatric patients were identified based upon ICD codes as VTE cases. Following manual review to verify appropriate case classification and restriction of the cohort to those with extant genotyping and European ancestry, there were a total of 109 cases. These were matched by age and sex to 448 European controls (Table 2). Multivariable logistic regression was performed with three PRS models. In the full 293‐SNP model, the PRS was associated with moderately increased risk (OR = 1.38, 95% CI 1.10–1.74, *p* = 0.003) of VTE after adjustment for age, sex, CVC placement, and cancer diagnosis (Table 4). History of a CVC insertion was the strongest predictor of VTE in the pediatric population (OR = 5.65, 95% CI 3.40–9.50, *p* < 0.001) (Figure 4). The 91‐SNP model demonstrated similar results, with a significant association between standardized PRS and VTE risk (OR 1.25, 95% CI 1.00–1.56, *p* = 0.04) and CVC placement remaining the strongest predictor (OR 5.46, 95% CI 3.29–9.13, *p* < 0.001, Table 4). Finally, a simplified 2‐SNP model incorporating only Factor V Leiden and Factor II variants did not demonstrate a significant association with VTE risk (OR 0.90, 95% CI 0.69–1.13, *p* = 0.409).

**TABLE 4:** Multivariable logistic regression of risk factors for venous thromboembolism (VTE) in pediatric population.

Variable	293‐SNP PRS OR (95% CI)	293‐SNP PRS *p* value	91‐SNP PRS OR (95% CI)	91‐SNP PRS *p* value
Age	0.99 (0.96–1.03)	0.85	0.99 (0.96–1.03)	0.76
Male	1.29 (0.81–2.06)	0.38	1.32 (0.84–2.11)	0.24
Presence of central line	5.65 (3.40–9.50)	< 0.001	5.46 (3.29–9.13)	< 0.001
Cancer diagnosis	0.84 (0.19–3.01)	0.99	0.78 (0.19–2.75)	0.72
Standardized PRS	1.38 (1.10–1.74)	0.003	1.25 (1.00–1.56)	0.04

*Note:* Two multivariable logistic regressions performed (one for 293‐SNP, one for 91‐SNP), adjusted for the first 10 principal components, age, sex, CVC insertion, and history of a cancer diagnosis.

Abbreviations: VTE, venous thromboembolism; PRS, polygenic risk score; OR, odds ratio; SNP, single nucleotide polymorphism; CI, confidence interval; SD, standard deviation.

Similar to findings in the adult cohort, the probability of VTE increased with increasing quantile of PRS score. For the lowest decile (< 10th percentile) of standardized PRS, there were 11 of 56 children (19.6%) with a VTE event. For the highest decile (> 90th percentile) of standardized PRS, there were 18 children (32.1%) with a VTE event (Table 1). Individuals in the top 10% of PRS distribution had 2.49 times higher odds of VTE compared to those in the bottom 10% (OR = 2.49, 95% CI: 0.98–6.33, *p* = 0.055), after adjusting for age, sex, CVC placement, cancer diagnosis, and principal components.

## 4 Discussion

This study utilized a large patient population with rich phenotypic and genotypic information to evaluate risk factors for VTE in adult and pediatric populations. In both the adult and pediatric populations, there was a moderate increased risk for VTE with increasing genomic risk with adjustment for known strong clinical risk factors. This correlates with prior findings in adults and provides evidence that whole‐genome genetic risk is also an important contributor to development of VTE in children. While polygenic risk for VTE has been previously analyzed in adult populations, this study utilizes three PRS models, incorporating a 293 variant model, more recent 91 SNP model, and simplified model incorporating only Factor V Leiden and Factor II variants and further demonstrates the efficacy of PRS for VTE in a pediatric population. In both cohorts, the 293‐SNP and 91‐SNP models demonstrated an increased association with VTE risk. The 2‐SNP model did not demonstrate a significant association with VTE risk in adults or children, suggesting that a more inclusive PRS captures broader genetic architecture beyond these two established thrombophilic variants alone. This reinforces and advances the evidence supporting broad genomic risk for VTE and offers a promising avenue for personalized prophylactic strategies across age groups [21].

While genomic risk for VTE does contribute to VTE incidence in children, the effect is significantly less than the clinical risk factor of CVC placement. These findings replicate prior studies demonstrating CVC as a strong risk factor for VTE, especially in children with cancer [29, 30, 31]. In our cohort, 15 of the 557 (2.7%) children had an oncology diagnosis and four of those 15 (26.6%) also had a VTE that developed after the cancer diagnosis. This certainly suggests the strong correlation of VTE in cancer patients who have a CVC. With the high need for CVC in both sick neonates and children in need of chemotherapy with cancer combined with anatomically smaller caliber vessels than adult populations, the high association of venous thrombosis in children with CVC was anticipated. The Children's Hospital Acquired Thrombosis consortium demonstrated that 80% of hospital‐acquired VTE in their registry were associated with a CVC [11]. Further, this strong association with CVC likely contributes to the lack of association of a cancer diagnosis with VTE in our cohort.

Following a CVC‐associated VTE in children, there is typically a recommendation for full anticoagulation to prevent the risk of recurrent thrombosis [32]. Prevention of thrombosis would be best; however, there are no defined criteria for prophylactic anticoagulation in children, especially neonates or children with a CVC [33]. However, if providers could identify those children with the highest risk of VTE, personalized approaches to anticoagulation utilizing clinical practice guidelines could be achieved. While the genomic risk was modest in comparison to the risk from CVC alone, knowing genetic risk profiles could provide opportunities for consideration of prophylactic anticoagulation. Particularly in pediatric patients for whom high‐quality data on prophylaxis for VTE prevention remain scarce, further work is needed to determine whether incorporation of PRS into future clinical risk prediction models improves risk stratification in pediatric populations [34].

The assembly of the PRS models in this study leveraged summary statistics from the UK Biobank, Copenhagen Hospital Biobank Cardiovascular Disease Cohort and The Danish Blood Donor Study, deCODE, Intermountain Healthcare, FinnGen, and the Million Veterans Program which afforded a notable advantage due to the size and homogeneity of the data [20, 22]. However, this data source presents clear limitations on the accuracy of the PRS among individuals of non‐European ancestry and future studies should seek to remedy this gap. Accordingly, this study was restricted to individuals of European ancestry, as differences in allele frequencies and genetic architecture across populations can reduce accuracy when a PRS is applied without recalibration. Future research should therefore prioritize validation of PRS models across non‐European and mixed populations to improve generalizability and equity in risk prediction. We also would like to determine if there are specific subcohorts of individuals in whom genetic risk affords higher propensity for VTE, for example those without other underlying risk factors, such as cancer diagnosis or CVC insertion, or if a more limited PRS would provide adequate risk prediction. We also did not incorporate the PRS into a formal clinical risk prediction model or assess discrimination metrics such as ROC curves or AUC. As such, we are unable to quantify the incremental predictive value of PRS beyond established clinical risk factors and acknowledge that additional clinical predictors contributing to VTE risk, such as diabetes and heart failure, were not captured in our analysis. Finally, the study cohort spans multiple years during which clinical practices and indications for interventions associated with VTE risk have evolved. We did not explicitly model calendar time, which may introduce residual confounding. Although we adjusted for available clinical risk factors, these may not fully capture changes in practice patterns over time.

The findings of this study hold significant implications for clinical practice in situations which introduce risk of VTE. Personalized risk assessment of genetic factors can guide clinical decision‐making regarding prophylaxis regimens. This is particularly important among pediatric patients, where there are currently no well‐defined criteria for either mechanical or chemo‐prophylaxis in children. Thus, identification of patients with high risk for VTE, using both genomics and clinical risk factors, could provide information to clinicians on which patients would benefit the greatest from prophylaxis. Further work is needed to determine whether incorporation of PRS into clinical risk prediction models improves risk stratification in pediatric populations.

## 5 Conclusion

This study introduces a novel approach to VTE risk assessment in both adults and children, integrating large quantities of genotypic information through a PRS combined with broad phenotypic information. With the known increasing incidence of VTE and a need to avert these complications, both clinical and genomic risk factors may be important to include in personalized prophylactic strategies for VTE prevention.

## Funding

Dr. McKay was supported by grant number T32 HS026122 from the Agency of Healthcare Research and Quality. The content is solely the responsibility of the authors and does not necessarily represent the official views of the Agency of Healthcare Research and Quality.

## Disclosure

The authors have nothing to report.

## Conflicts of Interest

The authors declare no conflicts of interest.

## Supporting information


**Table S1:** ICD9 and ICD10 codes for venous thromboembolism and pulmonary embolism phenotyping.

## Data Availability

The data that support the findings of this study are available on request from the corresponding author. The data are not publicly available due to privacy or ethical restrictions.
