# Supplementary material for: Harnessing Polygenic Risk Scores to Refine Venous Thromboembolism Risk Stratification
Source: Eur J Haematol. 2026 May 7;117(4):904–12. doi: 10.1111/ejh.70212 (PMC13160238; doi:10.1111/ejh.70212)
Supplement: Supplementary file 1 [file EJH-117-904-s001.docx]

**Supplementary Table 1:** ICD9 and ICD10 Codes for Venous Thromboembolism and Pulmonary Embolism Phenotyping

| **ICD 9/10 Code** | **Clinical Description of Code** |
| --- | --- |
| 453.0 | Budd-Chiari syndrome |
| 453.2 | Embolism and thrombosis of vena cava |
| 453.3 | Embolism and thrombosis of renal vein |
| 453.4 | Venous embolism and thrombosis of deep vessels of lower extremity |
| 453.40 | Venous embolism and thrombosis of unspecified deep vessels of lower extremity |
| 453.41 | Venous embolism and thrombosis of deep vessels of proximal lower extremity |
| 453.42 | Venous embolism and thrombosis of deep vessels of distal lower extremity |
| 453.82 | Acute venous embolism and thrombosis of deep veins of upper extremity |
| 453.84 | Acute venous embolism and thrombosis of axillary veins |
| 453.85 | Acute venous embolism and thrombosis of subclavian veins |
| 453.86 | Acute venous embolism and thrombosis of internal jugular veins |
| 453.87 | Acute venous embolism and thrombosis of other thoracic veins |
| 415.0 | Acute cor pulmonale |
| 415.1 | Pulmonary embolism and infarction |
| 415.13 | Saddle embolus of pulmonary artery |
| I82.0 | Budd-Chiari syndrome |
| I82.2 | Embolism and thrombosis of vena cava |
| I82.3 | Embolism and thrombosis of renal vein |
| I82.4 | Acute embolism and thrombosis of deep veins of lower extremity |
| I82.6 | Acute embolism and thrombosis of deep veins of upper extremity |
| I82.A1 | Acute embolism and thrombosis of axillary vein |
| I82.B1 | Acute embolism and thrombosis of subclavian vein |
| I82.C1 | Acute embolism and thrombosis of internal jugular vein |
| I26. | Pulmonary embolism |
